# Supplementary material for: Adolescents’ Perceptions of a Relapse Prevention Treatment for Problematic Gaming—A Qualitative Study
Source: Healthcare (Basel). 2023 Aug 22;11(17):2366. doi: 10.3390/healthcare11172366 (PMC10486974; doi:10.3390/healthcare11172366)
Supplement: Supplementary file 1 [file healthcare-11-02366-s001.zip › Interview guide for adolescents.pdf]

Interview guideline for adolescent

Research question:

- "How do young people who have undergone treatment for problematic computer gaming experience their treatment?"

Introduction:

- Thank you for agreeing to the interview

- Small talk

- Presentation of the interviewer

- Presentation of the purpose of the interview and what the data will be used for

- Tell approximately how long the interview will take

- Present the ethical guidelines according to the information letter and inform about the freedom not to answer all questions and the option to cancel the interview whenever you want

- Sign consent for the interview

- Ask if it is okay for the conversation to be recorded.

- "Is there anything you need or wonder about before the interview starts?"

Background

- How old are you?

- Did you go through the entire treatment?

- Do you play on a console or on a computer?

- Was it you or your parents who sought treatment for problem gaming

Theme: Young people's experience and understanding of problematic gaming

- Why do you play computer games?

- Has the reason why you play changed after the treatment?

- Do you usually play computer games with others?

    - If yes: In what setting do you usually play with others?

    - if no: Why not?

- What is problematic gaming according to you?

    - Have your understanding of problem gaming changed after you underwent the treatment?

        - If yes: How has your understanding of problem gaming changed after undergoing treatment?

- What do you find fun about your playing?
- What do you find boring about your playing?
- Do you think yourself that you have problematic gaming?
    - if yes: Can you tell us what you think is problematic about your gaming?
    - if no: Did you experience problem gaming before undergoing the treatment?

Theme: Problem gaming and your relationship with your parents

- How would you describe your parents' attitude towards your gaming?
    - Do you feel that their attitude has changed compared to before the treatment?
- Do you find that you argue a lot with your parents about your gaming?
    - if yes: What are you arguing about?
    - Do you experience any difference compared to before the treatment?
- Do you feel that your relationship with your parents has changed after the treatment?
    - if yes: in what way?
    - if no: why not?
- How would you describe your parents' understanding of why you play computer games?

Theme: Undergoing treatment for problem gaming

- What did you think of the treatment for problem gaming?
- What goals did you and your therapist set for the treatment?
- Did the treatment help you control your gaming?
    - if yes: what was most helpful?
    - if no: why not?
- What has been most helpful with the treatment?
- What has been least helpful about the treatment?
- Which part of the treatment did you find most helpful?
- Which part of the treatment did you find least helpful?
- How motivated were you to change your game before the treatment?
- How motivated are you to change your gaming now after the treatment?
- How did you experience your therapist's knowledge of computer games?
    - How did your therapist's knowledge of computer games affect your experience of the treatment?
- Has the treatment changed the way you understand and look at your computer gaming?

- How has the treatment changed the way you understand and look at your computer gaming?

- What did you think about the homework that you had to do between the sessions?

- Imagine that you are a therapist who is going to carry out a treatment against problematic computer gaming: What would you do differently?

Finish:

- Do you feel that you need more help with your playing?

- Do you know where to turn if you feel unwell?

- Is there anything else you would like to add regarding gaming or problematic gaming?
