# Supplementary material for: Adolescents’ Perceptions of a Relapse Prevention Treatment for Problematic Gaming—A Qualitative Study
Source: Healthcare (Basel). 2023 Aug 22;11(17):2366. doi: 10.3390/healthcare11172366 (PMC10486974; doi:10.3390/healthcare11172366)
Supplement: Supplementary file 1 [file healthcare-11-02366-s001.zip › Intervjumall for ungdomar.pdf]

Intervjumall för ungdomar

Forskningsfråga:

- "Hur upplever ungdomar som genomgått behandling för problematiskt datorspelande sin behandling?"

Introduktion:

- Tack för att du ställer upp på intervjun
- Småprat
- Presentation av intervjuaren
- Presentation av intervjuns syfte och vad data ska användas till
- Berätta ungefär hur lång tid intervjun kommer att ta
- Presentera de etiska riktlinjerna enligt informationsbrevet och informera om friheten att inte besvara alla frågor samt valmöjligheten att avbryta intervjun när man vill
- Skriva på samtycke för intervjun
- Fråga om det är okej att samtalet spelas in.
- ”Är det någonting du behöver eller undrar innan intervjun sätter i gång?”

Bakgrund

- Hur gammal är du?
- Genomgick du hela behandlingen?
- Spelar du på en konsol eller på en dator?
- Var det du eller dina föräldrar som sökte behandling för problematiskt spelande?

Tema: Ungdomarnas upplevelse och förståelse av problematiskt spelande

- Varför spelar du datorspel?
    - Har orsaken till varför du spelar förändrats efter behandlingen?
- Brukar du spela datorspel med andra?
    - Om ja: På vilket sätt brukar du spela med andra?
    - om nej: Varför inte?
- Vad är ett problematiskt spelande enligt dig?
    - Har din förståelse av problematiskt spelande förändrats efter att du genomgått behandlingen?
        - Om ja: Hur har din förståelse av problematiskt spelande förändrats efter att du genomgått behandlingen?
- Vad upplever du är roligt med ditt spelande?
- Vad upplever du är tråkigt med ditt spelande?

- Tycker du själv att du har ett problematiskt spelande?
    - om ja: Kan du berätta vad du tycker är problematiskt med ditt spelande?
    - om nej: Upplevde du att du hade ett problematiskt spelande innan du genomgick behandlingen?

Tema: Problematiskt spelande och relationen med föräldrar

- Hur skulle du beskriva dina föräldrars inställning till ditt spelande?
    - Upplever du att deras inställning förändrats jämfört med innan behandlingen?
- Upplever du att du bråkar mycket med dina föräldrar om ditt spelande?
    - om ja: Vad bråkar ni om?
    - Upplever du någon skillnad jämfört med innan behandlingen?
- Upplever du att din relation med dina föräldrar förändrats efter behandlingen?
    - om ja: på vilket sätt?
    - om nej: varför inte?
- Hur skulle du beskriva dina föräldrars förståelse för varför du spelar datorspel?

Tema: Att genomgå behandling för problematiskt spelande

- Vad tyckte du om behandlingen mot problematiskt spelande?
- Vilka mål satte du och din behandlare upp för behandlingen?
- Hjälpte behandlingen dig att kontrollera ditt spelande?
    - om ja: vad var mest hjälpsamt?
    - om nej: varför inte?
- Vad har varit mest hjälpsamt med behandlingen?
- Vad har varit minst hjälpsamt med behandlingen?
- Vilka övningar i behandlingen tyckte du var mest hjälpsamma?
- Vilka övningar i behandlingen tyckte du var minst hjälpsamma?
- Hur motiverad var du att förändra ditt spelade innan behandlingen?
- Hur motiverad är du att förändra ditt spelande nu efter behandlingen?
- Hur upplevde du din behandlares kunskap om datorspel?
    - Hur påverkade din behandlares kunskap om datorspel din upplevelse av behandlingen?
- Har behandlingen förändrar ditt sätt att förstå och se på ditt datorspelande?
    - Hur har behandlingen förändrat ditt sätt att förstå och se på ditt datorspelande?
- Vad tyckte du om hemuppgifterna som du skulle göra mellan samtalen?
- Tänk dig att du är en behandlare som ska genomföra en behandling mot problematiskt datorspelande: Vad skulle du göra annorlunda?

Avslutning:

- Upplever du att du behöver mer hjälp med ditt spelande?
- Vet du var du ska vända dig om du mår dåligt?
- Är det något annat du skulle vilja lägga till angående datorspel eller problematiskt datorspelande?
